# How Do Children Make Sense of their Parent’s Mental Health Difficulties: A Meta-Synthesis

**DOI:** 10.1007/s10826-018-1112-6

**Published:** 2018-06-19

**Authors:** Graham John Simpson-Adkins, Anna Daiches

**Affiliations:** Division of Health and Medicine, Lancaster University, Lancaster, LA1 4YG UK

**Keywords:** Parents, Children, Mental Health, Biopsychosocial, Qualitative

## Abstract

Children of parents who experience mental health difficulties (COPE-MHD) consistently demonstrate numerous negative outcomes, including risks of intergenerational continuity of mental health difficulties (MHD). Numerous studies have analysed the experiences and understanding of parents’ MHD from the perspective of COPE-MHD. This metasynthesis aims to capture, across available literature, the way in which COPE-MHD make sense of their parent’s MHD and how this perception impacts their life. For inclusion in the review, research articles were required to be published in peer-reviewed journals, apply qualitative methods of data collection and analysis and report on the direct accounts of COPE-MHD regarding their understanding or experience of their parents’ MHD. Five electronic databases were used; Academic Search Complete, CINAHL, MEDLINE, PsycINFO and Child Development and Adolescent Studies. Fourteen studies were included. Analysis produced three overarching themes. The findings illustrate children’s sophisticated biopsychosocial conceptualisation of the cause and process of their parent’s MHD. It also highlights how they utilise this understanding to manage the day-to-day concerns associated with their parent’s experience of MHD. Clinical implications highlight a need for services working with children, parents and families to more frequently enquire about parents’ MHD and to consider the outcomes of such enquiry in the psychological formulation of children and young peoples’ mental health and development. Limitations and recommendations for future research are presented.

Research has consistently indicated that parents’ mental health difficulties (MHD) can have major effects on children, leading to increased risk of behavioural, social, emotional and educational difficulties (Singleton 2007). Children of parents experiencing mental health difficulties (COPE-MHD) have also been found to be two to three times more likely to develop MHD compared to their peers (Cowling et al. 2004; van Doesum and Hosman 2009). Longitudinal findings from the USA, consistent at 10 and 20-year follow-up, revealed that children of parents with a diagnosis of depression were three times more likely to experience MHD associated with low mood and anxiety compared to their peers, with similarly elevated risk of developing substance dependence (Weissman et al. 1997, 2006).

The development of MHD in COPE-MHD has been associated with numerous risk factors and mechanisms, largely focussing on theories concerning genetic factors (Beardslee et al. 1983; Riley et al. 2003; Weissman et al. 2005). However, research has increasingly emphasised the importance of psychosocial factors (O’Connell 2008), including attachment relationships, parenting style and ability, socioeconomic factors and adverse life events, amongst many others (Fudge, Falkov, Kowalenko, & Robinson, 2004). COPE-MHD have been found to be at higher risk of numerous social adversities during childhood (Gladstone et al. 2011), such as increased risk of being placed in foster care (Kohl et al. 2011), experiencing mental health associated stigmatisation (Corrigan and Miller 2004) and experiencing parental suicide, which can all negatively impact child development (Göpfert et al. 2004). However, it is important to note that, despite increased risks of poor outcomes, many parents experiencing MHD parent effectively and many COPE-MHD do not experience any adverse effects (Smith 2004).

Most of the research concerning COPE-MHD has been quantitative and there have been calls for more qualitative approaches to this topic (Aldridge 2006; Sollberger 2007). Walsh (2009) suggested that, in order to understand the mechanisms linking parents’ MHD to negative outcomes in COPE-MHD, it is valuable to learn how COPE-MHD perceive their parents’ mental distress, what this understanding means to the children, and how this perception impacts upon their understanding of mental health. Currently, relatively little is known about what COPE-MHD understand about parents’ MHD. Much of the qualitative literature around the views of COPE-MHD has focussed on retrospective accounts of adult children (Baik and Bowers 2006; Foster 2010); reviewed by Murphy et al. (2011).

Gladstone et al. (2011) published a review of available literature on children’s experience of their parent’s MHD. However, their results do not illustrate how children use this knowledge to make sense of their parent’s MHD; the results primarily focus on children’s experience in terms of the impacts of their parent’s MHD on their daily lives. Furthermore, this review lacked transparency with regards to the search strategy and data analysis. This metasynthesis attempted to review and interpret the available literature to capture the current understanding, from the child’s perspective, about how COPE-MHD make sense of their parent’s MHD.

A preliminary literature search was conducted to help develop the research question and a comprehensive range of relevant search terms. The research question was defined as; “how do children understand their parents’ MHD?” For this review, MHD was defined as any psychologically-related distress of sufficient clinical severity to receive support from mental health service. This was done to avoid the need to rely on diagnostic terminology to determine inclusion.

## Method

### Search Strategy

The search took place in November 2015. Five electronic databases were used; Academic Search Complete, CINAHL, MEDLINE, PsycINFO and Child Development and Adolescent Studies. Due to a large return on search terms alone (>10,000), results were refined by using the age-range functions on Ebsco. The search was run with each applicable age range individually, one at a time; first by Childhood (birth-12 years), returning 1178 results, then “adolescence (13–17 years)” returning 992 results, then “school age (6–12 years)” with 765 results, “adolescence (13–18 years)” which returned 400 results, and finally “young adulthood (18–29 years)” which returned 811 results. These five sets of results were combined and searched together by accessing the search history on Ebsco, selecting each of these searches and then clicking on the “search with AND” function. The search results were restricted to research papers published in peer-reviewed journals, which functioned as pre-determined evidence of quality (Murray & Forshaw, 2013).

**Fig. 1:**
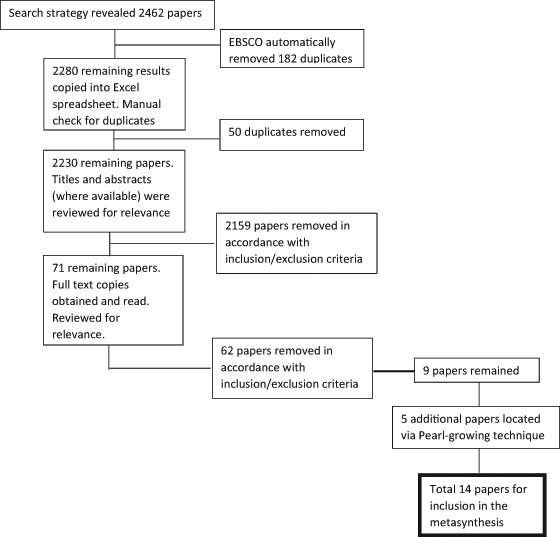
Flow diagram for inclusion of papers for the metasynthesis

The search returned 2,462 papers (see Fig. 1: Flow diagram for inclusion of papers for the metasynthesis). All papers selected for inclusion were published from 1992 to 2013 (see Table 1: characteristics of selected papers). The age range of one study (Griffiths et al. 2012) extended to 19. However, it was decided to include this study as it contained rich data principally within the selected age range for this review. The search results were then scanned for other papers that extended to 19 years; none were found. Two papers used the same sample and data. However, Mordoch (2010) conducted a secondary analysis on data collected by Mordoch and Hall (2008), to reanalyse the data for the purpose of answering a different question and as such, was not excluded.

**Table 1:** Characteristics of selected papers

Authors	Aim	Qualitative method	Sample	Setting
Webster (1992)	Assess the burden experienced by children of parents with a diagnosis of schizophrenia	Interviews, analysis not stated	20 children between 8–18 years (10 males, 10 females) of parents with a diagnosis of schizophrenia	Depot Injection Clinic. Manchester, UK
Garley et al. (1997)	Explore the subjective needs, cognitions and perceptions of asymptomatic children of parents with a mood disorder, to guide the development of a group intervention	Ethnographic approach, semi-structured focus groups, thematic analysis	6 children between 11–15 years (3 males, 3 females) of parent with a diagnosed mood disorder (depression and manic depression)	University-affiliated psychiatric facility. Toronto, Canada
Meadus and Johnson (2000)	Describe the experiences of adolescent children living with a parent who has a mood disorder	Unstructured interviews, Giorgi’s (1985) Descriptive Phenomenological method	3 female children aged 17 years living with a parent with a parent with a mood disorder	Psychiatric facility and volunteer organisation. Toronto, Canada
Handley et al. (2001)	(1) To identify the number of children of parents/carers with mental illness, (2) to identify the types of supports needed by parents, children and service providers (3) identify level of support available	Small groups and individual interviews	4 female children of parents with diagnosis of affective disorder, aged 11–15 years	Government Mental Health Services in the southern region of Tasmania, Australia
Riebschleger (2004)	Explore a child’s eye view of living day to day in a family that included a parent with a psychiatric disability	Secondary analysis of data from individual interviews and focus groups, Grounded Theory	22 children between 5–17 years (mean age = 9.36, 11 males, 11 females) of parents with a psychiatric disability	Prevention programs located in three community mental health agencies in northeast, southwest, and central Michigan, USA
Maybery et al. (2005)	Determine differences in perspective on issues facing children whose parents have a mental illness	Interpretative paradigm, separate child and parent focus group interviews, analysis not stated	12 children between 6–16 years of parents with mood disorder, personality disorder and psychotic disorder	North east Victoria, Australia
Cogan et al. (2005)	Explore the understanding and experiences of children affected by parental mental health problems	Semi-structured interviews, analysed using interactive model of Huberman and Miles et al. (1994)	20 children between 12–17 years (10 males, 10 females) of parents with IDC-10 diagnosis of an affective illness and 20 children between 13–17 years (10 males, 10 females) of ‘well’ parents	Recruited via family support workers. Glasgow, Scotland
Östman (2008)	Investigate experiences of children of parents with a severe mental illness	Thematic analysis	8 children between 10–18 years (3 males, 5 females) of parents with psychiatric diagnosis	Psychiatric unit. South of Sweden
Mordoch and Hall (2008)	Explore how children manage their experiences of living with a parent with a mental illness	Interviews, participant observation and drawings. Constant comparative grounded theory	22 children between 6–16 years living part of full-time with a parent with depression, schizophrenia or bipolar diagnoses	Midwestern Canadian city
Mordoch (2010)	Explore how children understand mental illness and what they want to tell other children living with parental mental illness	secondary grounded theory analysis of data from Mordoch and Hall (2008), focussed on investigation of a component of the ‘Monitoring’ category from the primary analysis	22 children between 6–16 years living part of full-time with a parent with depression, schizophrenia or bipolar diagnoses	Midwestern Canadian city
Venkataraman (2011)	Explore children’s perspectives on the parenting of mothers with a diagnosis of bipolar disorder	Initial semi-structured interview and follow-up interview. Constant comparative grounded theory	4 children between 10–15 years with mothers that had a diagnosis of bipolar disorder	Community mental health centres and support groups. Un-named town Midwest USA
Griffiths et al. (2012)	Explore the experiences of young people with a parent with obsessive compulsive disorder (OCD)	Semi-structured interviews. Inductive thematic analysis	10 children between 13–19 years (5 males, 5 females) with a parent with OCD	Mental health services and voluntary organisations, UK
Trondsen (2012)	Provide insight into the perspectives and experiences of children and adolescents living with a mentally ill parent	Action-oriented study of online self-help group for 2 years. Issue focussed analysis (Weiss, 1994)	16 adolescents between 15–18 years (1 male, 15 females) of parents with a mental illness using an online self-help group	Norwegian hospital-run self-help group
Van Parys and Rober (2013)	Explore how children experience parental depression and how they experience their own caregiving in the family	Family interviews. Thematic analysis	14 children between 7–14 years (5 males, 9 females) of parents hospitalised for depression	Psychiatric unit for affective disorders at University, Belgium

### Inclusion Criteria

Any published research article applying qualitative methods of data collection and analysis to study the perceptions of children and young people under 18 years of age regarding their understanding or experience of their parent’s MHD was considered relevant for inclusion. The upper age limit was selected based on the age limit for most children’s services in the UK (Division of Clinical Psychology 2015). There were no date restrictions. Studies were required to be published in English, due to resource restrictions. Issues have been raised regarding the reliability of many psychiatric diagnoses (Bentall 2004; Freedman et al. 2013); reliance on such constructs as descriptors of distress does not in itself clarify the experiences for either the parent or the child. For this reason, it was considered more inclusive to define MHD as any psychologically-related distress of sufficient clinical severity to receive support from mental health services. Therefore inclusion did not require any specific diagnoses.

### Exclusion Criteria

Studies including multiple perspectives, such as those of both children and parents were excluded if it was not possible to clearly and independently distinguish responses of children under 18 years. Similarly, studies applying mixed methods approaches were accepted for inclusion if the qualitative data were reported independently from the quantitative data. An additional exclusion criterion was applied during the reading phase of the review; studies that analysed children’s views following interventions aimed at improving the child’s understanding of MHD were removed if they did not include the child’s perceptions prior to intervention.

### Quality Appraisal of the Selected Papers

The Critical Appraisal Skills Programme (CASP) quality research checklist was used to evaluate selected papers. To express the strength of explanation of each CASP checklist question reported, a three-point scoring system was utilised to evaluate each paper (Duggleby et al. 2012); weak (1 point), moderate (2 points) and strong (3 points). A score of zero was given if the checklist item was not present in the paper. The scores ranged from four to 24 (Mean = 15.43, SD = 4.95). However, no papers were excluded based on quality appraisal to avoid papers containing rich, valuable data being discarded based on meticulous use of the CASP (Atkins et al. 2008). Rather, the CASP was used to weight the data within selected papers, based on methodology, study conduct and the utility and trustworthiness of the findings; higher scoring papers provided more influence on findings (Tong et al. 2012). For example, codes or quotes from papers with low CASP scores were only translated into themes or used to illustrate themes in the results if the content or concept of that code appeared similarly in papers with higher CASP scores (Table 2).

**Table 2:** CASP checklist results

	Research design	Recruitment strategy	Data collection	Reflexivity	Ethical issues	Rigorous data analysis	Clear statement of findings	Valuableness of the research	Total score
Webster (1992)	0	1	1	0	0	0	1	1	4
Garley et al. (1997)	3	3	3	2	2	1	2	2	18
Meadus and Johnson (2000)	3	3	2	1	3	2	3	2	19
Handley et al. (2001)	2	3	3	0	2	1	3	3	17
Riebschleger (2004)	2	3	3	1	0	2	3	2	16
Maybery et al. (2005)	1	2	2	0	1	1	1	2	10
Cogan et al. (2005)	2	3	3	1	3	3	3	3	21
Östman (2008)	2	3	2	1	3	2	2	2	17
Mordoch and Hall (2008)	3	3	3	1	3	3	3	3	21
Mordoch (2010)	3	2	1	1	0	2	3	3	15
Venkataraman (2011)	2	3	3	1	2	2	2	2	16
Griffiths et al. (2012)	1	2	1	3	1	3	3	3	17
Trondsen (2012)	3	3	3	3	3	3	3	3	24
Van Parys and Rober (2013)	3	3	3	1	2	3	2	3	20

### Synthesis of the Selected Papers

Results were analysed from an objective ontological stance; that there is a reality independent from human understanding. However, this stance is taken within a subjective epistemology; that knowledge of this reality stems from varying interpretations on that reality. These varying interpretations are developed via social interactionism, where “people strive and act toward what represents meaning for them”, where “meaning arises out of social interaction”, and “meaning is being dealt with and modified through interpretive processes” (Handberg et al. 2014, p. 1023).

The selected papers were synthesised using a meta-ethnography approach (Atkins et al. 2008; Noblit and Hare 1988; Reid et al. 2009). Papers were arranged and read in order of publication date, in order to position the research into a historical context. With each paper, we examined and reflected on developing codes by highlighting and extracting first and second order constructs; verbatim transcriptions of participant responses and subsequent interpretations by the authors of the paper being reviewed. This began the identification of emerging themes; extracts were re-read to produce new conceptualisations. Akin to a constant comparison approach, we attempted to continually translate emerging themes, allowing iterative emergence of reciprocal translations across papers (Reid et al. 2009). We examined and reflected on all preliminary themes, based on semantic and latent similarities, to develop preliminary key themes (Reid et al. 2009). Line of argument analysis helped relate and explain the preliminary key themes to develop the final key themes, or third order constructs, which signified the completion of the synthesis. Results were examined by all members of the research team to critique the conceptualisation of themes and concepts.

## Results

Papers selected for synthesis reported on parents with multiple mental health diagnoses, despite no requirement of this through inclusion criteria. Two papers reported on parents with diagnosed psychotic disorders, namely schizophrenia, six on mood or affective disorders, namely depression and bipolar spectrum disorders, one of parents with diagnosed obsessive compulsive disorder, one of parents with a diagnosed personality disorder, and three with a non-specified diagnosed “psychiatric disability” or “mental illness”. Interviewed children ranged from five to 19 years old, with highest clustering in teenage years.

Three overarching themes were developed. The first theme represents children’s conceptualisation of the cause and process of their parent’s MHD. The second describes how COPE-MHD manage their parents’ perceived vulnerability. The final theme illustrates children’s search for positive narratives, whilst managing the day-to-day difficulties presented by their parent’s MHD.

### Overpowered by the Physiological Consequences of Adversity: “Give us back my old mum”

This overarching theme captures how children seemingly view their parent’s MHD as primarily caused by mental distress associated with the parent’s experience of adversity, which leads to physical dysfunction and ultimately transforms the parent, if only temporarily. This process can result in a consequential sense of loss of the parent. This theme comprises three subthemes.

#### Adversity causes mental distress, which leads to physical dysfunction: “someone stuck a fork in, mushed it all up”

Over half of the papers reviewed described children’s views that MHD is triggered by environmental or psychosocial factors, particularly adverse or stressful life events. Cogan et al. (2005) described how 13 of the 20 interviewed children considered MHD as a consequence of painful or traumatic life events, such as ‘getting divorced’, ‘family arguments’, ‘grandpa…dying’ or ‘abuse’ (p.56). Children also appeared to put particular emphasis on the effects of childhood adversity as the cause of MHD.

It therefore appears that COPE-MHD seem to understand mental distress to be the result of difficult or traumatic experiences in life, suggesting a more psychosocial model of MHD. However, they also appear to consider that the consequences of such distress is the development of physical dysfunction. For instance, most papers highlighted children’s frequent use of medicalised language and interpretations of MHD as an ‘illness’, associated with a physically malfunctioning body or brain:

I’d describe a healthy brain as a freshly baked blueberry pie. You know everything is in its right place; it is all organized and ready to eat. A brain with a…Mental Illness…is a blueberry pie that somebody stuck a fork in, mushed it all up and everything is mixed up (Mordoch 2010, p. 23).

It was also frequently described that MHD requires treatment via specialist support such as from a medically trained professional, primarily involving medication. Beliefs about the need for medication were depicted well in the following quotes: “You won’t be able to talk to him if he’s not on his medication” (Mordoch and Hall 2008, p. 1131), and “my dad has to take all these tablets cos he’s not well, I think there’s something wrong with his brain” (Cogan et al. 2005, p. 56). This need for medication was often attributed to their parent somehow malfunctioning or being defective in some way, “the medication, the food, makes parents feel better because something is wrong with them” (Mordoch 2010, p. 23).

#### MHD takes over: “This isn’t my mum. She’s not acting right”

Children primarily appeared to be aware of MHD via observation of behavioural changes, such as increased duration and frequency of sleeping, not working and doing fewer household chores, losing interest in previously enjoyed activities, crying, shouting and unusual or inappropriate behaviours (Garley et al. 1997; Griffiths et al. 2012; Handley et al. 2001; Mordoch 2010; Riebschleger 2004; Trondsen 2012; Van Parys and Rober 2013). Children also recognise changes to their parent in terms of negative felt emotion, such as “getting angry more easily, and being sad” (Van Parys and Rober 2013, p. 334), and negative expressed emotion, such as “My mom starts yelling at me” (Riebschleger 2004, p. 57). Many studies reported that children described these changes as unstable and unpredictable, “sometimes he’ll get angry and sometimes he’ll be very nice. Like different…one minute later” (Garley et al. 1997, p. 101).

Most studies highlighted a view amongst children that their parent was separate from their MHD, with an understanding that these unpredictable changes were the result of the MHD and not a fundamental characteristic of the parent. For instance, Webster (1992) described how one child illustrated a sense that their mother was no longer the same person when she experienced MHD:

when she got ill we just wanted to get away. We didn’t want to know. She wasn’t our mum then…. I used to cry every night, you know. I used to pray to God at night and say, ‘Give us back my old mum. I don’t want this person. This isn’t my mum…She’s not acting right’ (p. 323).

COPE-MHD portrayed how MHD performs an almost possession-like subjugation of their parent, where the essence of the parent they knew before MHD, or at least when MHD are less pronounced, still fundamentally exists, but is not present when under the influence of MHD, “it’s just his illness, it’s not him talking.” (Mordoch and Hall 2008, p. 1134). This invasion presents a distorted version of their parent, “The problem is that I see the man, but not my father” (Tronsden, 2012, p. 181).

#### A sense of loss: “It was as though she did not exist”

All but one study (Van Parys and Rober 2013), described a sense of loss for these children. For example, there was a sense of emotional absence or unavailability of the parent, “She can’t be there emotionally for me” (Meadus and Johnson 2000, p. 386). There was also a sense of physical absence, “When she was ill she used to stay upstairs…she would live upstairs and we would live down here…It was as though she did not exist” (p. 320). Handley et al. (2001) portrayed how one child felt that MHD had “ripped” their parent from them (p. 225). Trondsen (2012) described a loss of parental interaction with the child, “I lack a mother…A mother who says something enthusiastically over the dinner table or asks and offers opinions, not just sits there and stares stone-faced into space.” (p. 181). Again, a quote in Trondsen (2012) highlighted the sense of loss and longing for the parent, despite them still being alive and present, “I often miss my dad so much that it hurts, even though he lives with me and I see him almost every day” (p. 181).

### Vulnerability, Protection and Secrecy: “always treading on eggshells”

The second overarching theme describes a consistent narrative across all papers that portrayed children’s attempts to alter their life in an attempt to protect their parent, themselves and life as they knew or desire it to be, driven by a perception that MHD renders their parent vulnerable. This theme consisted of three subthemes.

#### MHD are a persistent cause for angst: “I worry just in case”

All but one study (Maybery et al. 2005) described a sense of children feeling fearful and anxious about the effects of MHD on their parent and themselves, “My father’s illness mostly makes me scared. I hate being scared, but that is probably the feeling I’ve felt most during the last 12 years” (Trondsen 2012, p. 179).

Many children reported fears of losing their parent to hospitalisation or suicide, “I don’t think he’d [father] commit suicide. I don’t think he’d actually go to that level but sometimes I just worry just in case” (Mordoch and Hall 2008, p. 1135). Trondsen (2012) described how awareness of negative stereotypes can increase such fears, “I’ve heard many times lately about people with mental problems who have killed family members. My mom isn’t that bad now, but you never know” (p. 180).

Mordoch (2010) suggested how worries appear to increase due to the unpredictability and instability of changes in their parent’s behaviour and mood. Accordingly, children learned to monitor their parent for signs of change, as this enabled them to identify patterns of behaviour that symbolised MHD. Others described anxiously thinking about or checking on their parent due to a fear of self-harm or suicide, “when she was in the bathtub or if she had a razor or whatever. You just wanted to ask, Mom are you okay in there, or with whatever she was doing” (Meadus and Johnson 2000, p.386). Van Parys and Rober (2013) deduced that, due to an awareness or apprehension of parental suicide or harm, children can begin to interpret everyday behaviours, such as sleeping, as risky.

Finally, many studies described how children worried about MHD being transmitted to them, biologically or otherwise: “I was wondering if it was hereditary then, because if I felt down or something…then I was worrying, well am I going to be like that too.” (Meadus and Johnson 2000, p. 387). Trondsen (2012) described how some children can begin to monitor their own behaviour, as they do their parents, and associate emotions such as sadness as a sign of developing MHD.

#### Subjugate own needs for the parent’s: “Sometimes it’s more like I’m the one who’s the parent”

All but four of the papers described children attempting to adjust, poignantly described as “like always treading on eggshells” (Griffiths et al. 2012). This was done to avoid upsetting or burdening their parent, suggesting that the parent is viewed as “vulnerable” (Webster 1992, p. 321). Riebschleger (2004) exemplified such a response, “I try not to talk to her so that she doesn’t get upset and get worse. I try not to tell her things that would get her upset” (p. 27). Maybery et al. (2005) stated that children described needing to “be quiet when their parent was unwell” (p. 6). Other attempts not to burden or upset the parent included doing housework, caring for siblings, avoiding provocation, not bringing friends home, getting out of the house or generally staying away from the parent (Mordoch and Hall 2008; Mordoch 2010; Venkataraman 2011; Trondsen 2012; Griffiths et al. 2012). It was also found that children hide their worries from their parents and reassure them that they were not affected by their parent’s distress (Van Parys and Rober 2013).

Children appear to view their parent as in regular need of help, support and comfort as a result of MHD. For example, many of the papers described children increasing their responsibilities in order to support their parent, “I do all the washing and ironing. I just help out really wherever it needs…if she’s ill I’ll do the whole house” (Griffiths et al. 2012, p. 74). A perception that the parent needs to be taken care of and comforted was depicted in Venkataraman (2011), “Parents need help and children can talk to them, try and understand what is wrong: Sometimes you have to take care of your parent” (p. 23). Some of the studies depicted how children recognised a change to their role in the family, particularly parentification, “Sometimes it’s more like I’m the one who is the parent, and he is the child. I feel like he’s my responsibility, and that I have to take care of him” (Trondsen 2012, p. 181).

#### A family secret: “It’s not something we talk about”

Children seem to hold a belief that, as a result of MHD, their parent or family is not “normal” (Östman 2008, p. 357). They are acutely aware that, socially, the subject of mental health is often taboo, meaning MHD are often treated as a “family secret” (Riebschleger 2004, p. 28), due in part to an apparent awareness of stigmatisation from others. For instance, Östman (2008) described how children recognised that others, including neighbours or distant relatives, sometimes perceived their family as different from “normal” families. In Riebschleger (2004), two of the children portrayed being treated differently by proxy after being taken into care, “They (foster parents) treated me like I was some kind of delinquent or something. I didn’t do anything wrong.” (p. 25).

Children subsequently develop a desire not to reveal the family secret, “it’s just like a problem that’s in the home. You don’t want to let it out” (Griffiths et al. 2012, p. 75). This message sometimes appears to be encouraged by parents, “My parents tell me to keep it hush-hush.” (Mordoch and Hall 2008, p. 1134). Three papers drew attention to a sense of embarrassment due to their parent’s MHD, inferred as another reason why it is kept as a family secret (Handley et al. 2001; Griffiths et al. 2012; Webster 1992).

### Searching for a Needle of Hope in a Haystack of Adversity: “You don’t know what’s going on and it’s hard to be happy”

It appears throughout that COPE-MHD perceive their parent’s MHD as something which is unpredictable and confusing, but that is nevertheless an accepted, albeit difficult part of their life. They are impacted in many negative ways, but there is evidence of an active attempt to reformulate these from a positive, resilience-building perspective. The final overarching theme illustrates a struggle by COPE-MHD to develop alternative, positive narratives about their parent, family life and their own ability to cope as a means of surviving and making sense of the direct and indirect adverse psychological impacts of their parent’s MHD. This theme consisted of two subthemes.

#### The impact of persistent uncertainty: “I hide myself in my room, and feel deeply sad”

All papers depicted practical and psychological difficulties experienced by COPE-MHD as a result of exposure to and attempts to manage the unpredictability of their parent’s MHD. Negative impacts were often associated with a sense of overwhelming burden faced by children, due to increased responsibilities when the parent is experiencing MHD:

I will stay at the house all day because I don’t want anyone to walk in or anything and I can’t lock the door because my sister is already gone and she doesn’t have her key. It…just feels like I have lot of responsibilities sometimes…sometimes it is just like wow! (Venkataraman 2011, p. 101).

Östman (2008) inferred that “children experience great suffering in taking responsibility when no one else does” (p.356). Van Parys and Rober (2013) suggested that children reflect on the impact of MHD on their lives and can consequently feel down or become more easily angered. Trondsen (2012) further exemplified this negative emotional impact on children:

It is really hard coming home after school and unexpectedly finding my mum…sitting in her chair, depressive, unkempt and tousle-headed, staring expressionless at the television, but without attention, and not saying a word…I hide myself in my room, and feel deeply sad (p. 179).

Mordoch and Hall (2008) further exemplified the impact on children’s mental health, “I actually remember questioning what I was doing in my life. I wonder if I should kill myself. Everything was like a blur. You don’t know what’s going on and it’s hard to be happy.” (p. 1141).

#### The search for a silver lining: It's “not all bad, you know”

Despite difficult experiences, children also described feeling “used to it” (Griffiths et al. 2012, p. 76). It was unclear whether there was a sense of hopelessness or acceptance in relation to these comments, but their experiences were described as “just part of my life” (Meadus and Johnson 2000, p. 387).

Cogan et al. (2005) suggested that COPE-MHD were less likely to express stigmatising opinions of MHD generally, due to personal experience of living with someone experiencing MHD. Some actually described positive attitudes towards mental health as a consequence of living with their parent’s MHD, “I am not concerned because it makes me who I am and it makes me a unique kind of person” (Venkataraman 2011, p. 103). However, it appeared from some of the comments that children were actively attempting to develop alternative, positive narratives about their parent and the meaning they attribute to the impact of the parent’s MHD. For instance, Griffiths et al. (2012) described one child’s attempt to develop an alternative narrative, by suggesting that experiencing their parent’s MHD had helped them to learn helpful ways to manage their own mental health, “I know I’ll always have [the worries] but I’m going to learn ways just like my mum does of handling them” (p. 76).

## Discussion

This metasynthesis is an attempt to combine and analyse qualitative data from 14 studies, with the aim of providing a functional conceptualisation regarding the sense children make of their parent’s MHD. Results suggest that children appear to cite environmental or psychosocial factors, primarily traumatic experiences and particularly those occurring in childhood, as the main causes of their parent’s MHD. However, they view that psychological distress stemming from these adversities triggers an illness comprising physical dysfunction. They appear to believe that this illness subsequently overcomes the parent, and maintain that any changes to the parent’s behaviour or mood are a consequence of the MHD, and not a fundamental characteristic of the parent. Viewing MHD as an illness coincides with a view that the parent therefore requires specialist support, particularly medical support and chiefly medication. This need for specialist support and the perceived behavioural and emotional ‘abduction’ of the parent by MHD is understood to mean that the parent is ‘abnormal’ and highly vulnerable. Children consequently make numerous lifestyle alterations to protect this vulnerability, namely via a dominant and perpetual worrying, burdensome parentification and the harbouring of the family secret to fend off stigmatisation. The consequence of these alterations seemingly results in detrimental psychological impacts on COPE-MHD, despite their efforts to develop alternative, preferred narratives that paint their parent and their ability to cope in a positive light. It is hypothesised that this search for alternative narratives reinforces and is reinforced by the belief that the parent is out of control when under the influence of MHD. This conceptualisation and theorised process will require further investigation to confirm its clinical functionality.

### Theoretical and Clinical Implications

This concept of MHD as physical dysfunction, resulting from psychosocial adversity, which consequently requires specialist support, may have implications for how COPE-MHD make sense of their own responses to adversity and the subsequent support they may seek. For instance, as in Meadus and Johnson (2000), findings suggested that COPE-MHD self-monitor and subsequently associate their experiences, such as feeling sad, as a sign of developing MHD, based on their experience of their parent and the sense they make of their parents’ MHD. It was also found that children experience intense psychological distress in terms of frequent worries about their parent. Negative inferences about MHD, such as the parent being vulnerable and socially unusual, may support the development of problematic assumptions, or schemata, regarding mental health generally. Schema development has been previously associated with cognitive risk factors for the intergenerational continuity of MHD in COPE-MHD (Yehuda and Bierer 2008). This finding demonstrates a need to continue and expand on efforts to raise public understanding of mental health, particularly for children and young people, including via the provision of mental health-awareness training, such as Mental Health First Aid (Kitchener et al. 2013; Hadlaczky et al. 2014). These psychological impacts may also be of particular interest to researchers exploring mediating and moderating factors for the development of MHD for COPE-MHD. This review may add value to such research by providing an insight into possible cognitive processes involved, resulting from the way COPE-MHD make sense of mental health via experience of their parent’s MHD.

The awareness of social stigma supports previous research which suggests that relatives experience associative or courtesy stigma (Angermeyer et al. 2003; Koschade and Lynd-Stevenson 2011). This review also highlighted that COPE-MHD experience shame and isolation resulting from their awareness of courtesy stigma, initiated in part by familial requests for secrecy, which also supports previous findings (Pitman and Matthey 2004; Polkki et al. 2004). In response, children consequently avoid inviting friends home, experience embarrassment and feel unable to communicate their experience to others for fear of humiliation. COPE-MHD experience or fear indirect social stigmatisation as a consequence of their relationship to their stigmatised parent, which has a subsequent disabling effect on self-expression and certain social behaviours. It appears, therefore, that COPE-MHD may experience a form of secondary psycho-emotional disablism (PED) (D. Reeve, personal communication, April 18, 2016) as a result of courtesy stigma, which may indicate another possible mechanism of intergenerational continuity. PED is a form of social oppression operating at the private level, enacted via, for instance, restrictions on ways of being resulting from the stigmatising messages of others or the internalisation of such messages, resulting in internalised oppression (Reeve 2015; Thomas 2007). PED can destabilise one’s sense of self and self-esteem (Reeve 2008, 2015), the psychological impacts of which have been compared to that of emotional abuse (Reeve 2006). This possible phenomenon may have implications for the development of MHD for COPE-MHD and requires further investigation.

Walsh (2009) proposed that children who consider MHD as external to their representation of their parent may be more likely to have better outcomes than those that embed MHD, particularly the negative aspects, within their mental representation of their parent. The ability to externalise MHD from the parent or self has been associated with greater resilience in COPE-MHD (Beardslee et al., 2003; Walsh 2009). The discovery from this review that children appear to externalise MHD from the parent, may suggest the utility of narrative therapy approaches with COPE-MHD, in order to support this process of externalisation (Daniel and Wren 2005; Focht and Beardslee 1996; Pluznick and Kis-Sines 2014). Furthermore, this review supports findings that personal exposure to MHD can promote positive attitudes towards people with MHD (Angermeyer & Matschinger, 1996). A narrative therapy approach may also support COPE-MHD to capitalise on these positive attitudes and help to move them from problem saturated understandings of mental health, to more preferred stories (White and Epston 1990), which may encourage the development of helpful, adaptive schemas.

The experience of reported psychological distress by COPE-MHD found in this review may encourage those caring for or supporting a child to consider their parents’ mental health as a systemic contributing factor in child presentations of anxiety or low mood. However, it has been suggested that COPE-MHD may not demonstrate observable behavioural difficulties during episodes of increased parental distress and are thus unlikely to be known to services (Cooklin 2010). The desire not to burden the parent, also identified in this review, may mean that children’s distress goes unnoticed or undisclosed until more severe. Weir and Douglas (1999) suggested that COPE-MHD can often appear superficially fine until ensuing disclosure highlights experiences of adversity. Furthermore, retrospective accounts of adult COPE-MHD highlight a desire to receive more information about MHD, alongside professional support for themselves during childhood (Knutsson-Medin et al. 2007). This may highlight a need to develop procedures for routine enquiry, such as that described by McGee et al. (2015), which encourages enquiry about various adverse childhood experiences, of which having a parent that experiences MHD is a well-recognised item (Chapman et al. 2004).

The above discussion should be considered within the typical limitations of qualitative reviews. For example, the analytic quality of the review is dependent in part on study quality. However, papers were not excluded based on quality appraisal to reduce the risk of excluding valuable interpretive data that could obstruct the development of new conceptualisations. Moreover, the search strategy was restricted only to papers published in peer-reviewed journals, excluding alternative sources, such as grey literature. The intention of this decision was to support the systematic quality and transparency of the search and to increase the ease of replication, as well as to ensure a minimum standard of research included. It is however acknowledged that additional relevant qualitative research may therefore have been excluded, which may have effected findings. Another limitation is that third order constructs presented are dependent on the reliability and rigor with which first and second order constructs were developed by the authors in the reviewed papers (Duggleby et al. 2012). However, in agreement with Murray and Forshaw (2013), these findings could nevertheless be considered robust, as the presented themes were developed across literature that varies in terms of context, sample characteristics, publication date and study setting.
